# Vertical ground reaction force oscillation during standing on hard and compliant surfaces: The “postural rhythm”

**DOI:** 10.3389/fneur.2022.975752

**Published:** 2022-09-01

**Authors:** Stefania Sozzi, Manh-Cuong Do, Marco Schieppati

**Affiliations:** ^1^Istituti Clinici Scientifici Maugeri IRCCS, Centro Studi Attività Motorie (CSAM), Pavia, Italy; ^2^Complexité, Innovation, Activités Motrices et Sportives (CIAMS), Université Paris-Saclay, Orsay, France; ^3^Complexité, Innovation, Activités Motrices et Sportives (CIAMS), Université d'Orléans, Orléans, France

**Keywords:** vertical ground reaction force (VGRF), frequency spectra analysis, vision, support surface, body sway

## Abstract

When a person stands upright quietly, the position of the Centre of Mass (CoM), the vertical force acting on the ground and the geometrical configuration of body segments is accurately controlled around to the direction of gravity by multiple feedback mechanisms and by integrative brain centres that coordinate multi-joint movements. This is not always easy and the postural muscles continuously produce appropriate torques, recorded as ground reaction force by a force platform. We studied 23 young adults during a 90 s period, standing at ease on a hard (Solid) and on a compliant support (Foam) with eyes open (EO) and with eyes closed (EC), focusing on the vertical component of the ground reaction force (VGRF). Analysis of VGRF time series gave the amplitude of their rhythmic oscillations (the root mean square, RMS) and of their frequency spectrum. Sway Area and Path Length of the Centre of Pressure (CoP) were also calculated. VGRF RMS (as well as CoP sway measures) increased in the order EO Solid ≈ EC Solid < EO Foam < EC Foam. The VGRF frequency spectra featured prevailing frequencies around 4–5 Hz under all tested conditions, slightly higher on Solid than Foam support. Around that value, the VGRF frequencies varied in a larger range on hard than on compliant support. Sway Area and Path Length were inversely related to the prevailing VGRF frequency. Vision compared to no-vision decreased Sway Area and Path Length and VGRF RMS on Foam support. However, no significant effect of vision was found on VGRF mean frequency for either base of support condition. A description of the VGRF, at the interface between balance control mechanisms and sway of the CoP, can contribute information on how upright balance is maintained. Analysis of the frequency pattern of VGRF oscillations and its role in the maintenance of upright stance should complement the traditional measures of CoP excursions in the horizontal plane.

## Introduction

During upright stance, postural muscle activations are distributed in time and space and produce the forces that move and brake the body segments, because our bipedal stance is unstable. The resultant dynamics of those local movements are recorded as ground reaction force by the platform upon which subjects stand (1, 2). The output of the platform sensors is often exploited to reconstruct the instantaneous positions of the Centre of Pressure (CoP), which is represented in the horizontal plane and is characterized by the length of its excursion (Path Length), the area of the surface covered (Sway Area) and the frequency content of its motion (3–6).

The body oscillates in an unpredictable way. During the natural standing posture, the Centre of Mass (CoM) is normally in front of the ankle joint and a gravity-induced torque caused by the imperfect correspondence of the CoP and CoM positions accelerates the body forward. This is opposed partly by the intrinsic ankle joint stiffness [(7–12), see (13, 14)] and partly by corrective actions of the postural muscles (15–17). These act onto the upper and lower body masses and produce interacting torques about the hip, knee and ankle joints, thereby maintaining the body motion within a region much smaller than the boundary of the base of support (18–23).

Standing upright implies control of both the vertical and the horizontal accelerations of the CoM. Any change in the CoP-CoM distance, which originates from muscle activities that make the joints/segments rotate, produces an imbalance broken down into horizontal and vertical torques. The CoP displacement relative to the CoM projection will create positive and negative horizontal torques allowing to maintain postural balance. The dynamic characteristics (frequencies and magnitudes) of the CoP displacements should be found in the horizontal forces. This is probably a reason why most studies on postural control were only interested in the CoP excursions. While the effective benefit of having exact knowledge about the values of the CoP displacement in the horizontal plane only is still being discussed [(4, 24–27); see (28)], little attention has been devoted to the vertical component of the ground reaction force (VGRF). This is certainly not the same as the body weight, but can vary around that value depending on the action of the postural muscles counteracting gravity. Not all the effects of the production of torques by the postural muscles can give rise to changes in the CoP-CoM relationships in the horizontal plane. Conversely, these horizontal changes may even conceal some interesting features of the neural control that appear in the VGRF.

Therefore, in this preliminary study, we addressed the characteristics of the VGRF modulation, following prior work that considered VGRF oscillations along with the CoP displacement in the description of the mechanisms underpinning standing posture (29–31). Being aware of some features of VGRF could contribute to enhance our insight on how balance is controlled and maintained, since the mechanical effect of the VGRF variations on the physics of the standing task have not been fully elucidated.

Here we compared the amplitude and frequency characteristics of the VGRF between vision and no-vision and standing on a hard floor vs. a foam pad, two conditions known to enhance body sway. We analyzed the VGRF in healthy young volunteers standing upright and leveraged the spectral analysis of the VGRF oscillations to help explain the effect upon postural stability of support surface features and vision. We also compared amplitude and frequency of the VGRF with the geometric measures of the CoP excursions. The effect of vision was analyzed in some detail, because vision selectively modulates the frequency of the CoP sway in certain frequency ranges (4, 32), but its effect on the VGRF features is still poorly understood (29, 33). On the other hand, the compliant support surface would magnify the VGRF and allow contrasting the VGRF under conditions requiring a different effort.

When healthy young subjects stand quietly on the bare force platform, the body sway is limited to within a small surface area and little muscular activity is present (34), while standing on a compliant surface, such as a foam pad, is obviously different (4, 35–39) and highly effective in terms of CoP excursions. Subjects continuously control and correct their stance by exerting a greater effort compared to standing on hard ground (40, 41), where the low level, almost-tonic activity of the muscles about the ankle can normally restrain the CoM from falling forwards. On foam, the elastic properties of the compliant support would in some way modify the correcting actions by acting as a damped spring (42), and could produce slower oscillations. Hence, the amplitude of the VGRF oscillations is expected to vary more than on hard ground and their variability would be larger as a consequence of the effort of standing on the challenging support (37). Conversely, the frequency of the VGRF oscillation would be higher on hard than compliant support, because small-amplitude and frequent correcting reactions brought about by our multi-segmented body would suffice to counteract the CoM sway (20, 43). Different frequencies of VGRF oscillations with or without vision and on solid or compliant support would be signs of distinct balancing strategies employed to cope with modifications in sensory and support conditions (44).

## Methods

### Participants

Twenty-three healthy young adults (12 females and 11 males, age 28.8 ± 4.3 years old, height 171.6 ± 5.9 cm, and weight 65.6 ± 11.8 kg) participated in this study. They had no history of neurological or musculo-skeletal disorders and had no sight problems or their visual acuity was corrected. No subject reported injuries or occurrences of falls in the previous year. All participants gave written informed consent according to the Declaration of Helsinki principles, which was approved by the local review board (Istituti Clinici Scientifici Maugeri IRCCS, approval number #2564-CE). The analysis was partly performed on previously recorded data (5) and partly on new data recorded in additional volunteers.

### Procedures

Subjects were asked to maintain upright stance for 100 s on a force platform (Kistler 9286BA, Switzerland) with the outer profiles of the parallel bare feet at hip width. Subjects performed one trial in different visual and base of support conditions: eyes closed (EC) and eyes open (EO) with Solid (the force platform) or Foam support. Vision and base of support conditions were randomized across subjects and performed in different days (at least 1 day elapsed between conditions). In the Solid condition, the rigid platform was topped by a thin linoleum lamina. In the Foam condition, a standard pad (Airex Balance Pad, Switzerland; L 50 cm, W 41 cm, H 6 cm, density 55 g/dm^3^, Young's module 260 kPa) was placed onto the platform (45). The outline of the feet was marked on a paper sheet fixed on the top of the platform or on the foam pad. Subjects stood at ease (46, 47) and looked at the structured visual scene of the laboratory wall at 6 m distance (4, 48) with both Solid and Foam support. During the trials, subjects would avoid major movements of the upper body and head (in pitch, roll and yaw). All subjects were naive to foam-standing. The last 90 s-epoch of each 100 s stance trial was acquired (49), in order to exclude the adjusting phase occurring after stepping onto the platform or on the foam pad. None of the subjects lost balance while standing on Foam despite the increase in sway compared to Solid support (4). There was no obvious effort at maintaining balance, without displacing the feet, as by flexing the trunk (23) or by performing upper arm movements, as reported by the experimenters sitting at a short distance from the platform and observing the subjects during the acquisition. No subject ever lifted a foot or made a step, either.

### Data acquisition and processing

The platform data, from which the ground reaction force was obtained and the CoP excursion computed, were acquired at the sampling frequency of 140 Hz by the SMART Capture software (BTS, Italy). Post-acquisition analysis was done using Excel (Microsoft, USA), LabVIEW (National Instrument, USA) and Origin (OriginLab Corporation, USA). The vertical component of the vertical ground reaction force (VGRF) and the CoP excursions along the anteroposterior (AP) and mediolateral (ML) directions were high-pass filtered at 0.01 Hz and low-pass filtered at 10 Hz with a fourth order Butterworth filter, after removing the respective mean values, with a software developed in LabVIEW. The length of the path (Path Length) was the total length of the time series (90 s) of the CoP displacement in the horizontal plane. Sway Area was the surface of the 95% ellipse fitted to the dispersion of the time-series data on the horizontal plane (50). The same LabVIEW software was used to calculate the Root Mean Square (RMS, the “effective value” of the waveform) of the VGRF for each subject, vision and support condition as a measure of the amplitude of the VGRF oscillations during the acquisition epoch.

The frequency analysis of the VGRF was performed by means of the Auto power spectrum Virtual Instrument (VI) algorithm of the LabVIEW functions. This VI calculated the fast Fourier transform of the VGRF time-series of each trial, subject, visual and support condition. The VI produced a single-sided power spectrum (the positive half of the frequency spectrum from 0.01 to 70 Hz). The resolution (sample frequency/sample number of the VGRF signal) was 0.011 Hz for the sampling frequency of 140 Hz (4) and a sample number of 12,600 (=90 ^*^ 140).

The power spectrum profile of the VGRF had the shape of a normal distribution function curve (see Results, **Figure 3**). Hence, for each participant and for each condition we fitted the VGRF spectrum profile with a Gaussian function, $y\;=\;Ae^{-\frac{{\left(f-\mu \right)}^{2}}{2\sigma^{2}}}$ by means of the "Curve Fitting” analysis tool of the software Origin, where A indicates the amplitude of the Gaussian function (i.e., the peak value of the curve corresponding to the mean value of the VGRF oscillation frequency), *e* is the Euler number, μ represents the frequency (*f* ) at which the peak in the power spectrum profile occurs (henceforth the Mean frequency) and σ is the Standard Deviation (SD) of the Gaussian function. For each experimental condition, the goodness of the fit was estimated by the Pearson's R coefficient. A Gaussian distribution curve was also fit to the mean spectrum profile obtained by averaging all individual subjects' spectra for each condition.

In order to detect differences between base of support conditions, the values of the VGRF RMS, Mean frequency, SD of the Gaussian fit and Peak amplitude of each subject were plotted for Solid vs. Foam support. The relationship between Mean frequency and Peak amplitude of the VGRF oscillations was also studied by a linear regression model. All vision and support conditions collapsed, the relationship between Mean frequency and Peak amplitude of the VGRF oscillations showed a hyperbolic trend (see Results, **Figure 6**). These data were fitted with a homographic function: $y\;=\;\frac{mx}{k\;+\;x}$ by means of the iterative conjugate gradient method of the Excel Solver Utility.

The relationship between the VGRF oscillation frequency and the geometric measures of the CoP displacement was assessed by plotting the values of Sway Area and Path Length of each subject against the corresponding values of the Mean frequency calculated on the Gaussian fit of the VGRF spectrum. These relationships were studied by a linear regression model and the R^2^ was calculated. A linear regression model was also used to detect any relationships across subjects between the amplitude of the VGRF RMS and the geometric measures (Sway Area and Path Length).

Assumptions for parametric statistics, evaluated by the Kolmogorov-Smirnov and Levene's test, were met for all the variables of interest. The effects of the different visual and base of support conditions on VGRF RMS, VGRF Mean frequency, VGRF Peak amplitude, SD of the Gaussian fit, Path Length and Sway Area were compared by 2 (Solid or Foam) × 2 (vision conditions) repeated measure (rm) ANOVA. Where the differences were significant, the effect size $\eta_{\text{p}}^{2}$ (partial eta squared) was reported. *Post-hoc* analysis was performed using the Fisher's LSD test. The significance level was set at 0.05. The minimum detectable effect size was computed using the G^*^Power sample size calculator (51). Given the number of our participants (*n* = 23), the study proved to have a sufficient power (>80%) to detect an effect size (Cohen's *d*) of 0.53. Statistical tests were performed using Statistica (Statsoft, USA).

## Results

### The amplitude of the VGRF oscillations during stance

**Figure 1:**
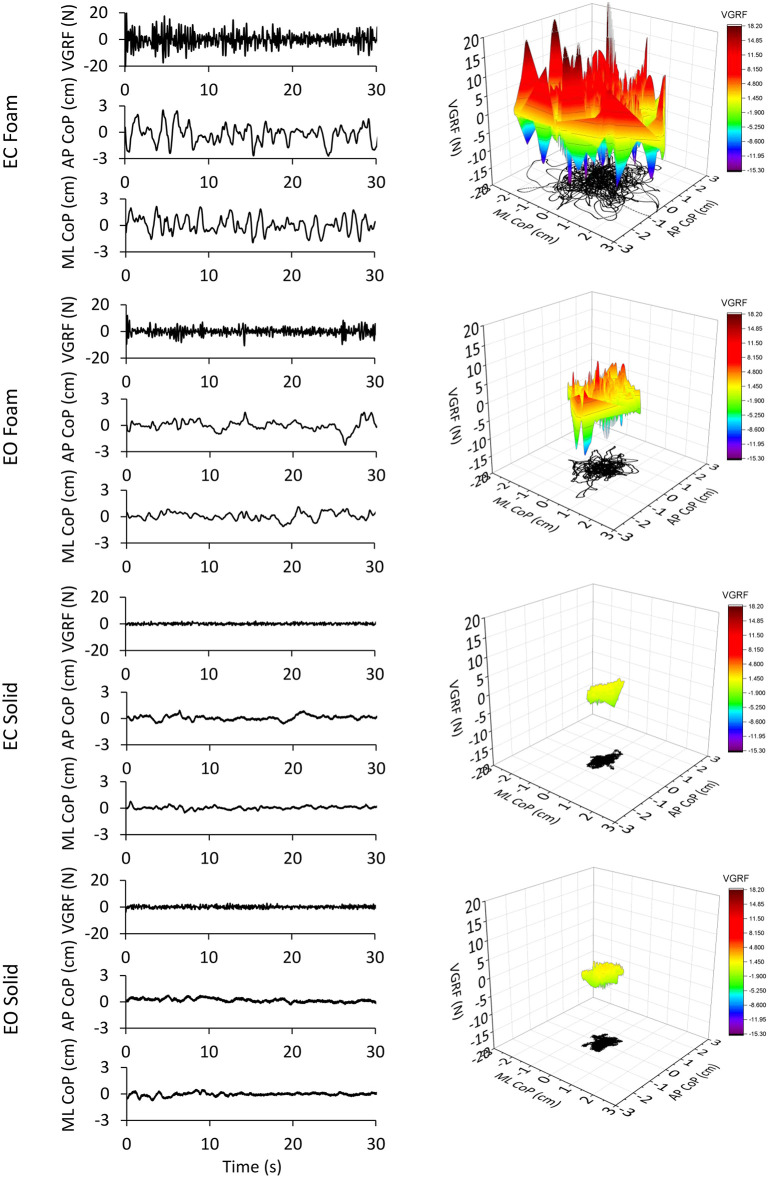
VGRF variations and CoP displacement in a representative subject. In the left column are reported the VGRF oscillations around the mean value (i.e., the subject's weight) and the CoP oscillations around the mean position for both ML and AP directions in the four different experimental conditions (eyes closed, EC or eyes open, EO) with Foam base of support and EC or EO with Solid base of support). Only the first 30s of the 90s acquisition epochs are shown. In the right side of the Figure, the VGRF oscillations are plotted against the CoP displacements on the horizontal plane in the four conditions of interest. VGRF oscillations and CoP displacements were the greatest in the EC Foam condition. With EO Foam, VGRF oscillations and CoP displacements diminished and became the smallest with Solid base of support where vision had little effect.

The values of the time-series of the VGRF of a typical subject are depicted in Figure 1, together with the corresponding time series of the CoP excursions along the ML and AP directions. The four trial conditions are represented. An epoch of 30 s only (selected from the first part of the entire acquisition epoch to distinguish the signal features) has been shown for clarity of representation of the data time-series out of the entire trial duration (90 s). It appears from the upper panel of the left column of Figure 1 (EC Foam) that the VGRF values range between about −20 N and +20 N (changes with respect to the body weight) and the CoP excursions between −1.8 and 2 cm (ML) and −2.7 and 3 cm (AP). Moreover, the VGRF and the CoP signals have a remarkably different frequency content (compare in each panel the top with the middle and bottom traces). The EC conditions feature a larger amplitude of the noisy trace of the VGRF and larger excursions of the CoP oscillations.

In the right column of Figure 1, the VGRF values are reported on the vertical axis, with their positive and negative values, their amplitude being referred to the color calibration bar on the right. On the horizontal plane, the corresponding point-to-point instantaneous CoP positions in the AP and ML axes are reported in black. The CoP excursions were small and occupied a surface area similar in the EO and EC conditions when standing on Solid support. Sway moderately increased in the EO Foam condition and was the largest in the EC Foam condition. During the 90 s acquisition epoch, the VGRF varied continuously around its mean value. VGRF oscillation amplitudes were much larger on Foam, less so with EO than EC, and similarly small on Solid support.

**Figure 2:**
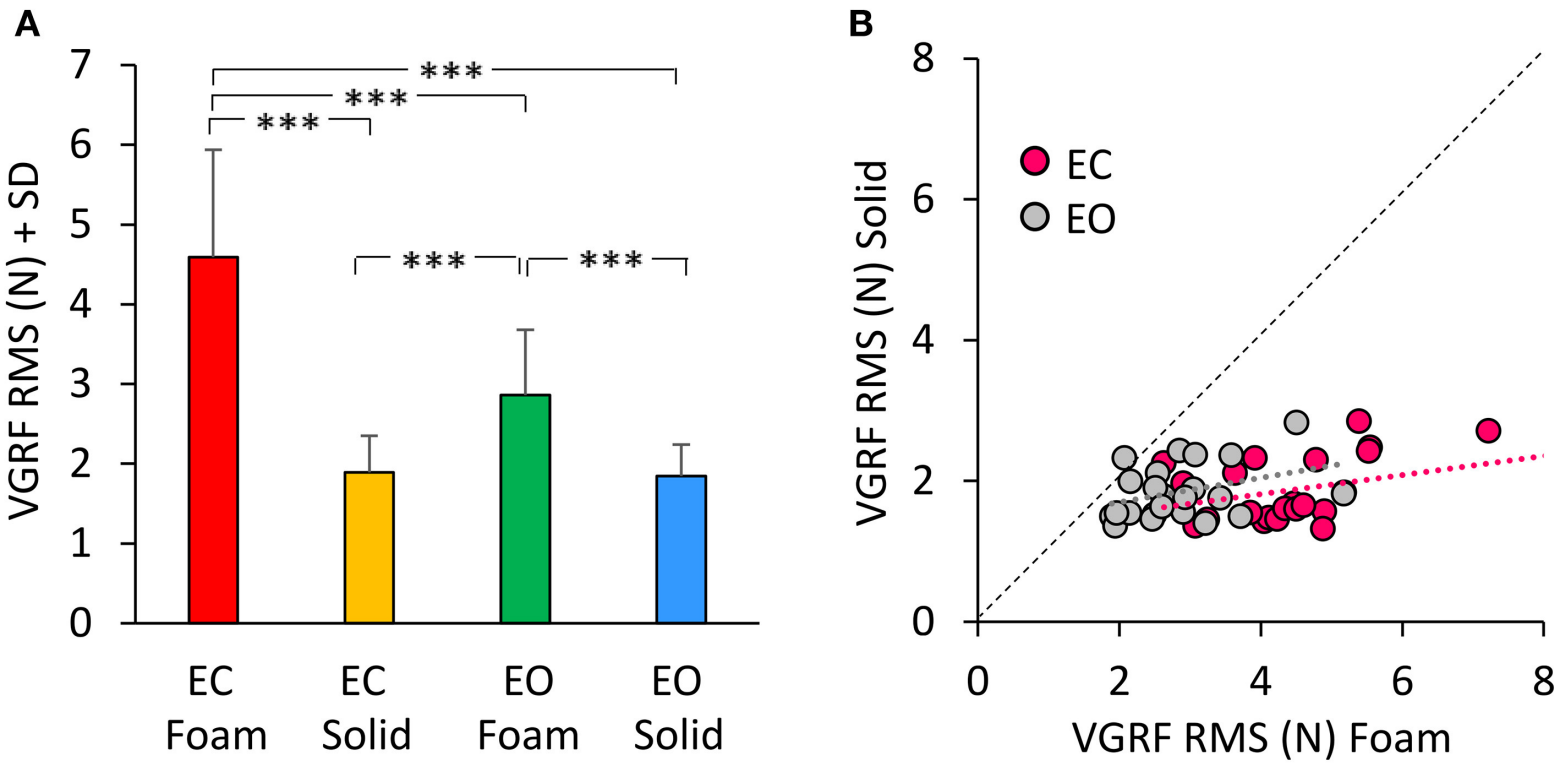
The VGRF Root Mean Square (RMS). **(A)** The mean values of VGRF RMS are shown for the two vision conditions (eyes closed, EC; eyes open, EO) and for the two bases of support (Foam and Solid). **(B)** The values of VGRF RMS for Solid are plotted against those for Foam, for each subject and vision condition (EC, pink dots; EO, gray dots). The black dotted line is the identity line. The VGRF RMS were greater with Foam than Solid under both vision conditions. The equations of the regression lines fitting the Solid vs. Foam data were: EC, y = 0.14 x + 1.27 (R^2^ = 0.16, *p* = 0.06); EO, y = 0.17 x + 1.35 (R^2^ = 0.13, *p* = 0.09). Asterisks indicate significant differences (***p < 0.001).

Figure 2A shows the mean values of the VGRF Root Mean Square (RMS) calculated across all subjects as a comprehensive measure of the amplitude of the VGRF oscillations over time for each visual and support condition. The VGRF RMS was the largest on the EC Foam condition and decreased in the order EC Foam > EO Foam > EC Solid ≥ EO Solid. There was a difference between Foam and Solid support [Foam > Solid, main effect, *F*_(1, 22)_ = 114.9, *p* < 0.001, $\eta_{\text{p}}^{2}$ = 0.84]. With EC, the RMS of the VGRF oscillations were greater than with EO [main effect, *F*_(1, 22)_ = 37.16, *p* < 0.001, $\eta_{\text{p}}^{2}$ = 0.63]. There was also an interaction between vision and support conditions [*F*_(1, 22)_ = 43.86, *p* < 0.001, $\eta_{\text{p}}^{2}$ = 0.66], because the difference between EC and EO was significant with Foam (*post-hoc, p* < 0.001) but not with Solid support (*p* = 0.79). In Figure 2B, the VGRF RMS data of all the subjects are plotted Solid vs. Foam. The graph shows in detail the much lower RMS of the oscillations on Solid (ranging from 1 to 2 N in the ordinate) than on Foam support (mainly ranging from 2 to 6 N in the abscissa). Almost all dots lie below the diagonal (where Solid = Foam). It also shows that removal of vision (pink dots) increased the RMS values compared to EO (gray dots) in most subjects on Foam but not on Solid support (the mean values of the gray and pink dots were different on the horizontal but not on the vertical axis). The regression lines fitting the Solid vs. Foam data were not different between visual conditions (*F*-test: slopes, *p* = 0.81; intercepts, *p* = 0.24).

### The probabilistic distribution of the frequencies of the VGRF oscillations

**Figure 3:**
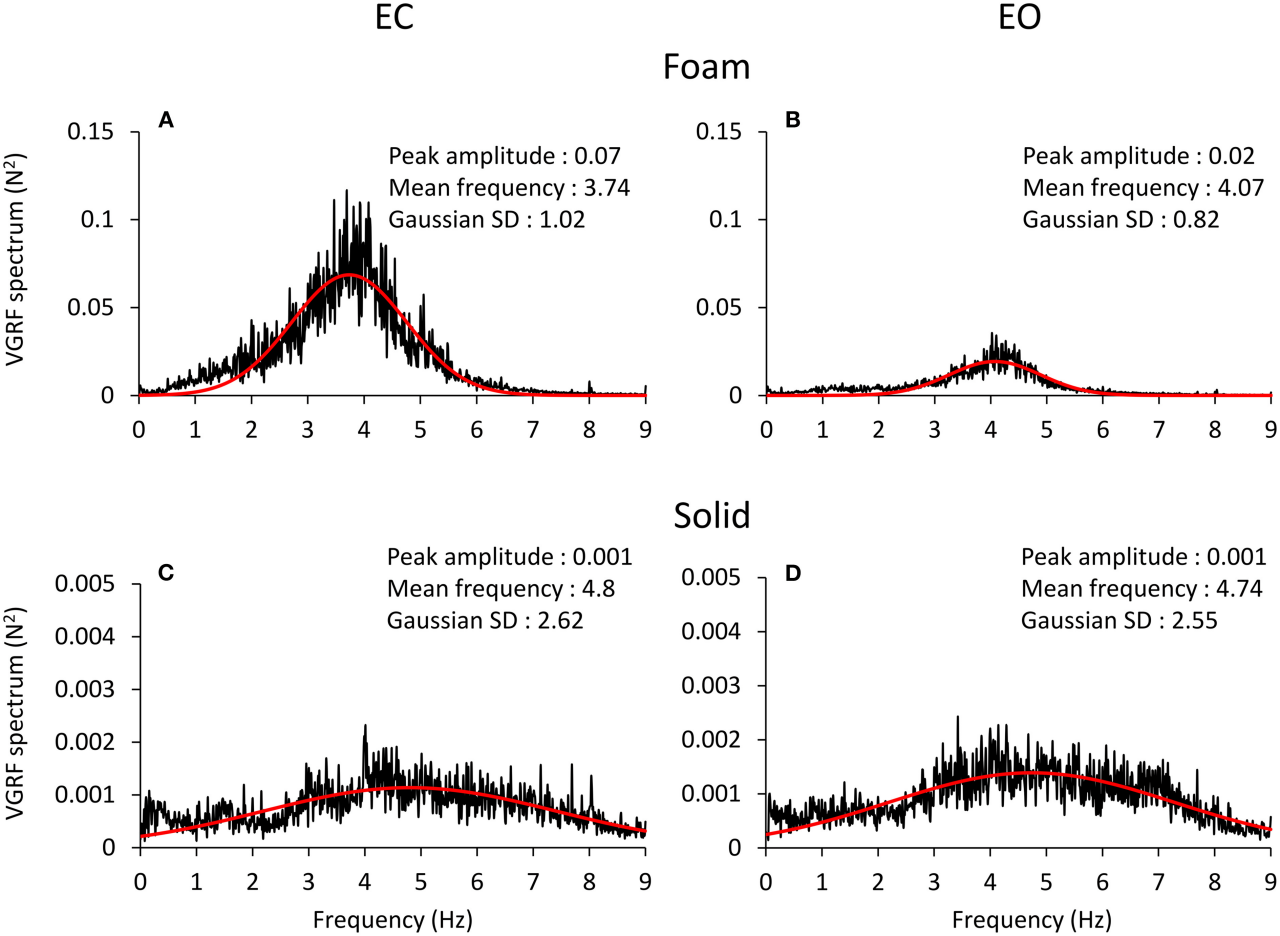
VGRF frequency spectrum. The mean spectrum profiles across subjects are reported for EC Foam **(A)**, EO Foam **(B)**, EC Solid **(C)** and EO Solid **(D)** conditions. The VGRF spectrum profile shows a bell curve-like shape, fitted with a Gaussian (red) curve. The parameters of the curve fitting the spectrum are reported in each panel. The VGRF spectrum had a much greater amplitude with Foam than Solid support. Note the extended scale in the ordinates of **(C,D)**, due to the peak amplitude of the spectrum with Solid support being less than one tenth of that with Foam **(A,B)**. The VGRF spectrum had a much greater amplitude with Foam than Solid support. The effect of vision was unambiguous with Foam support only. The frequency at the peak of the Gaussian fit was around 4 Hz with Foam and around 5 Hz with Solid support. The Standard Deviation (SD) of the Gaussian fits were larger for Solid than Foam support.

The spectral profiles of the oscillations in the VGRF time series showed frequencies across a relatively ample range independently of vision and support conditions (Figure 3). The profiles had the shape of a normal distribution curve, where the frequencies at both the left and the right side of the peak had very low spectral amplitude. In all four conditions, the profiles featured a peak at an oscillation frequency around 4 Hz.

The four panels of Figure 3 show the spectrum profiles, computed as the average of the spectra of all the subjects, with EC (left) and EO (right), on Foam (A and B) and on Solid ground (C and D). The more represented frequencies occurred in a relatively short range, between 3 and 6 Hz, regardless of vision and surface conditions. The overall amplitude of the spectra (note the different scale in the ordinate) was remarkably different in the order EC Foam > EO Foam > EC Solid ≥ EO Solid. The peak values of the Gaussian curves fitting the mean spectrum profile are reported in each panel. The shape of the curves reflected a strong tendency for the VGRF oscillation frequencies to gather around a common value, regardless of the characteristics of support and vision. The frequency of the spectrum at the peak of the Gaussian curve is referred to here as the Mean spectral frequency. There were relatively large standard deviations of the Gaussian curves, larger for the Solid (the curves were flat and broad) than the Foam support.

**Figure 4:**
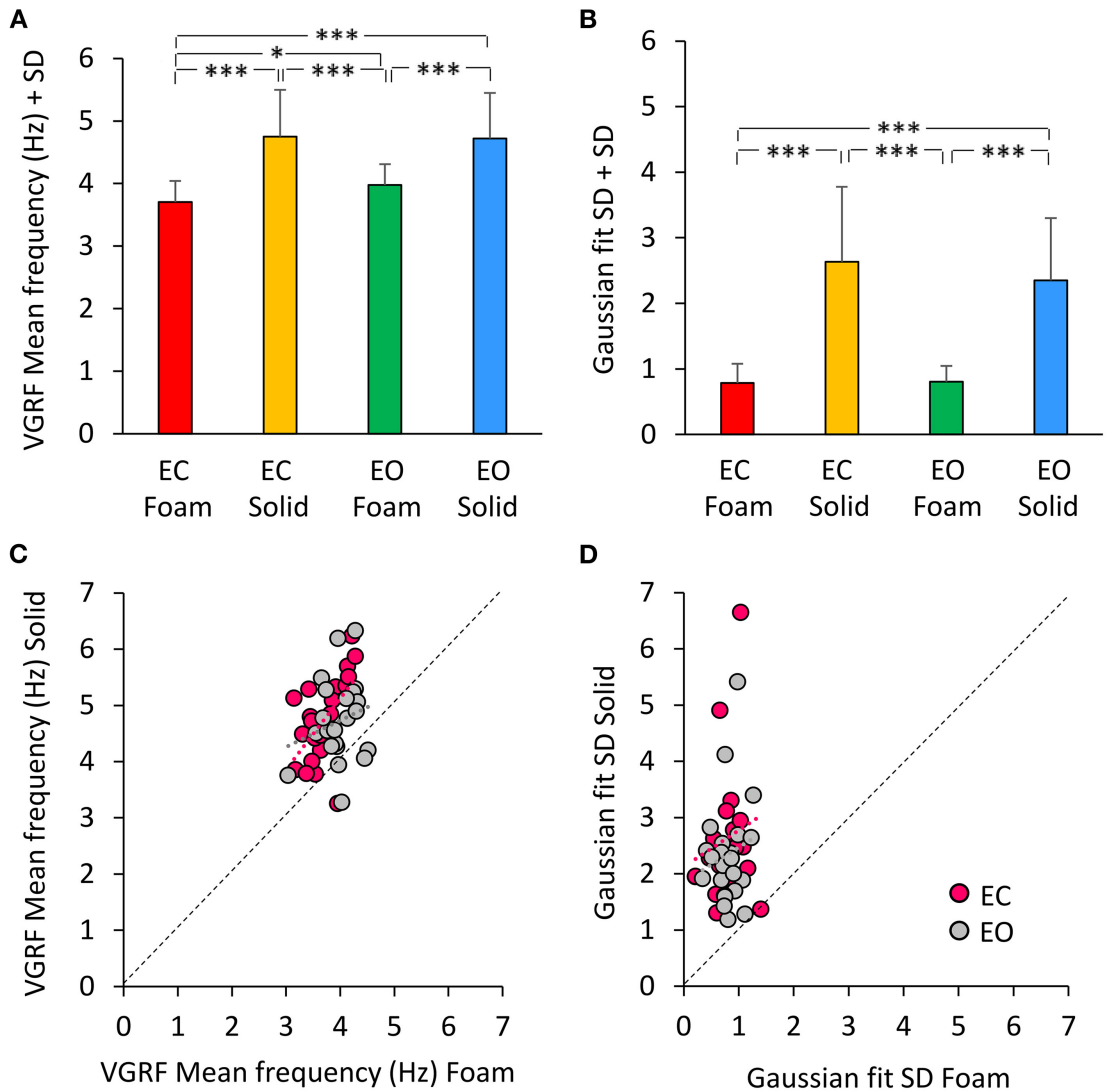
The VGRF mean frequency and the SD of the Gaussian fit. The mean values of the VGRF Mean frequency **(A)** and of the SD of the Gaussian curve **(B)** calculated across subjects are reported for each visual and support condition. The relationship between the Solid and Foam support data for the Mean frequency values and SDs are shown in **(C,D)**. Pink dots are referred to EC, gray dots to EO condition. The black dotted lines are the identity lines. The Mean frequency and the SD were greater for Solid than Foam support. The dot distribution in the abscissa shows that regardless of the support condition, there was no difference between EC and EO in VGRF Mean frequency and SD. The equations of the lines fitting the Solid vs. Foam Mean frequency values were: EC, y = 1.26 x + 0.06 (R^2^ = 0.32, *p* < 0.01); EO, y = 0.47 x + 2.84 (R^2^ = 0.05, *p* = 0.33). The regression lines fitting the Solid vs. Foam SDs of the Gaussian fit were y = 0.65 x + 2.12 (R^2^ = 0.03, *p* = 0.44) for EC, and y = 0.61 x + 1.86 (R^2^ = 0.02, *p* = 0.48) for EO. Asterisks indicate significant differences (*p < 0.05; ***p < 0.001).

A Gaussian curve was then fit to the spectrum profile of each participant in the different vision and base of support conditions. For each power spectrum profile, the goodness of the fit was estimated by the Pearson's R coefficient. The mean value of R was about 0.6 for the Foam conditions (with both EC and EO) and about 0.3 for the Solid conditions (EC and EO). Figure 4A shows a summary of the Mean frequency values across subjects for each vision and support condition. There was a difference in Mean frequency between support conditions [main effect, Solid > Foam, *F*_(1, 22)_ = 52.9, *p* < 0.001, $\eta_{\text{p}}^{2}$ = 0.71], but not in Mean frequency between visual conditions [main effect, *F*_(1, 22)_ = 2.74, *p* = 0.11]. The interaction between visual and support conditions was significant [*F*_(1, 22)_ = 4.54, *p* < 0.05, $\eta_{\text{p}}^{2}$ = 0.17], because there was a difference between EC and EO condition on Foam (*post-hoc, p* < 0.05) but not on Solid support (*p* = 0.78). Figure 4B shows the mean standard deviations (SD) of the Gaussian curves across subjects. The mean SDs were similar to those obtained by fitting the normal curve to the averaged spectra. The mean SD was greater for Solid than Foam support [main effect, *F*_(1, 22)_ = 110.4, *p* < 0.001, $\eta_{\text{p}}^{2}$ = 0.83] under both EC and EO conditions. There was no significant difference between visual conditions [main effect, *F*_(1, 22)_ = 0.76, *p* = 0.39] and no significant interaction between visual and base of support conditions [*F*_(1, 22)_ = 1.09, *p* = 0.31]. In Figure 4C, the individual values of the Mean frequency are plotted for Solid against Foam support condition, and the vision state is indicated by the colored dots (EC, pink; EO, gray). Across the subjects, for the Solid support (ordinate), frequencies ranged from about 3 to 6.5 Hz, whereas for Foam support the prevalent frequencies were comprised in a narrower range, from about 3.0 to 4.5 Hz, with a relatively large scatter across subjects. Reference to the identity line confirms that the prevailing frequencies of the VGRF oscillations had a higher value for Solid than Foam support in most subjects regardless of vision condition. Vision compared to no-vision produced a minor increase in the frequencies on Foam but not on Solid support. The regression lines fitting the Solid vs. Foam data were not different between visual conditions (F-test, slopes *p* = 0.8, intercepts *p* = 0.37). Figure 4D shows the values of the SDs of the Gaussian curves in each subject with Solid plotted against Foam condition. SDs were comprised in a larger range for Solid than Foam across subjects. The regression lines fitting the Solid vs. Foam SDs of the Gaussian fit were not different between visual conditions (slopes *p* = 0.8; intercepts *p* = 0.37).

### Amplitudes of the mean values of the VGRF oscillation spectra across vision and support surface conditions

**Figure 5:**
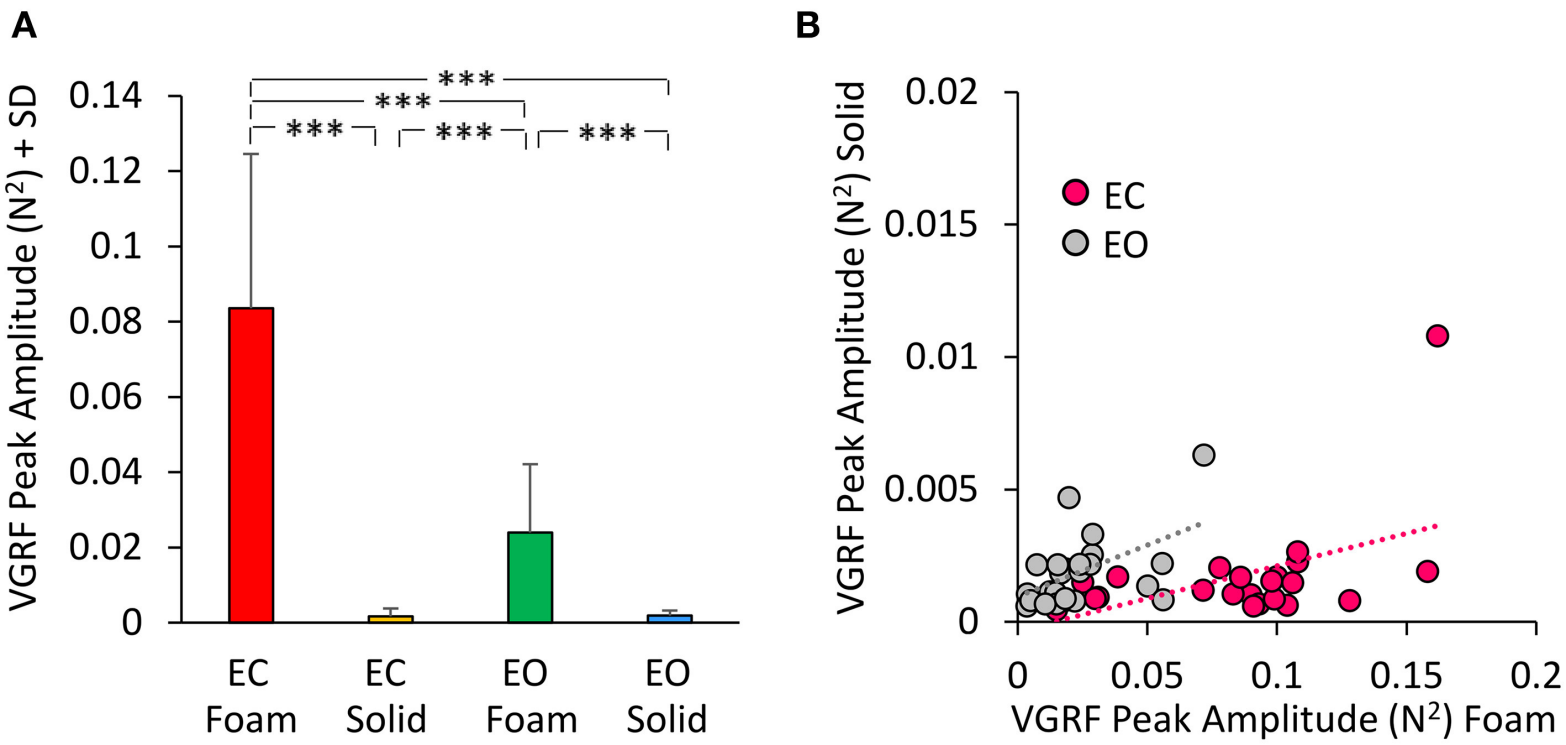
VGRF Peak amplitude. The mean VGRF Peak amplitude **(A)** are reported for each vision and support condition. Amplitude was the greatest in EC Foam condition and decreased with EO Foam. It was the smallest with Solid support, where vision had no effect. **(B)** The VGRF amplitudes for Solid are plotted against those for Foam, for each subject and vision condition (EC, pink dots; EO, gray dots). The equations of the lines fitting the Solid vs. Foam data points were: EC, y = 0.024 x- 0.0003 (R^2^ = 0.23, *p* < 0.05); EO, y = 0.039 x + 0.001 (R^2^ = 0.26, *p* < 0.05). Asterisks indicate significant differences (***p < 0.001).

The Peak amplitude calculated on the Gaussian fit is a measure of the amplitude of the VGRF oscillation at its Mean frequency of oscillations. The peak of the curves indicates a strong prevalence of the spectral amplitudes around 4 Hz. We analyzed these amplitudes in Figure 5. The mean peak values of the VGRF spectrum (N^2^) are reported for each vision and support surface condition. The Peak amplitude of the VGRF spectrum was small on Solid support (both EC and EO), increased in the EO Foam condition, and was the largest in the EC Foam condition (Figure 5A).

Differences were significant between support conditions [Foam > Solid: main effect, *F*_(1, 22)_ = 87.36, *p* < 0.001, $\eta_{\text{p}}^{2}$ = 0.79] and between visual conditions [EC > EO, main effect *F*_(1, 22)_ = 79.16, *p* < 0.001, $\eta_{\text{p}}^{2}$ = 0.78]. There was a significant interaction between visual and support conditions [*F*_(1, 22)_ = 84.34, *p* < 0.001, $\eta_{\text{p}}^{2}$ = 0.79]. *Post-hoc* analysis showed a difference between EC and EO with Foam (*p* < 0.001) but not with Solid support, where both Peak amplitudes were quite low (EC = EO, *p* = 0.97). In Figure 5B the Peak amplitude of the power spectrum on Solid base of support was plotted against the amplitude of power spectrum on Foam for EC (pink dots) and EO conditions (gray dots) for all subjects. The regression lines fitting the Solid vs. Foam data had a similar slope (*p* = 0.62), but different intercepts (*p* < 0.05).

**Figure 6:**
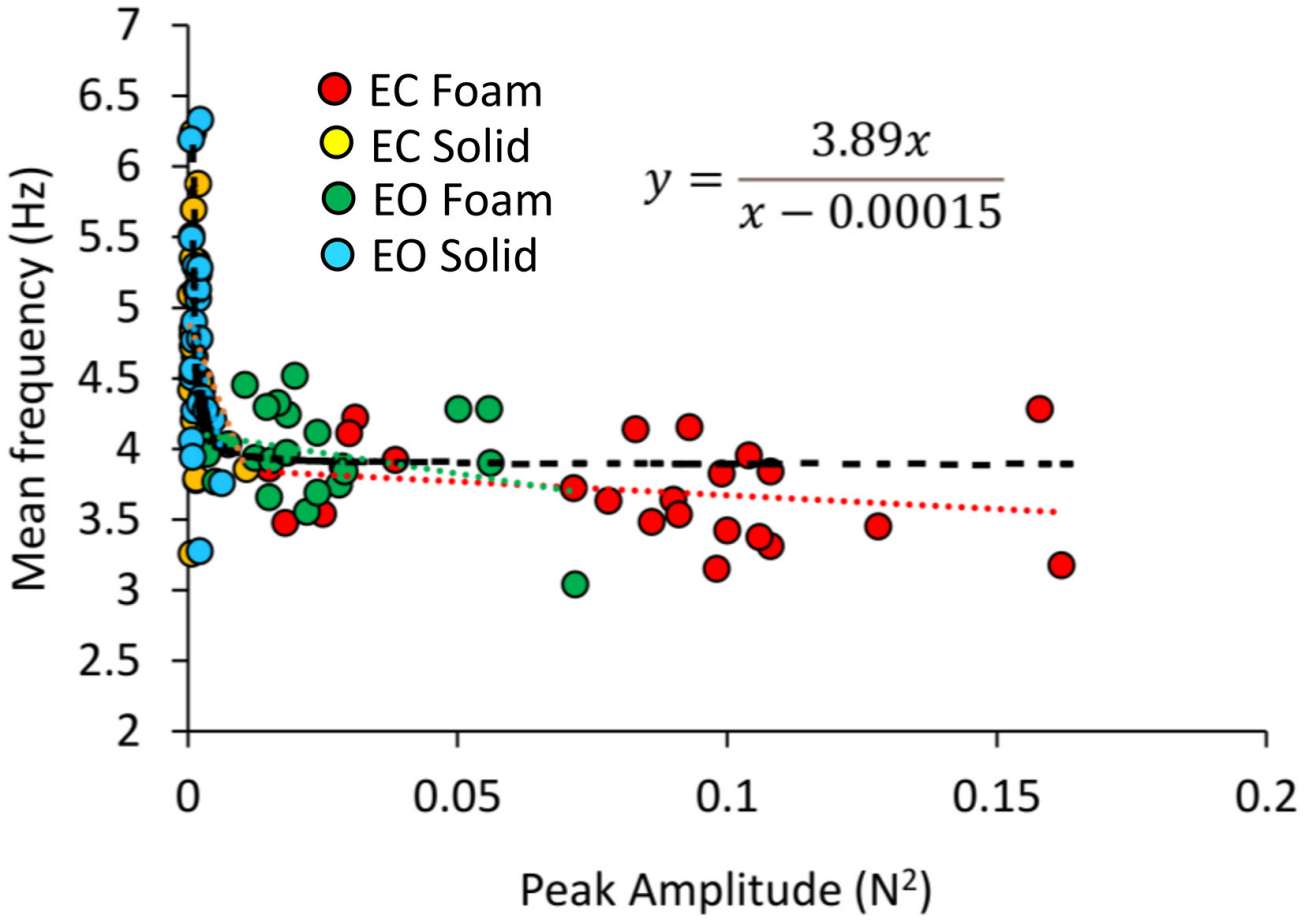
Relationship between Mean frequency and Peak amplitude of the VGRF spectrum. For each subject, the values of the Mean frequency are plotted against those of the Peak amplitude of the Gaussian curve in the different visual and base of support conditions (EC Foam, red; EC Solid, yellow; EO Foam, green; EO Solid, blue). The data distribution showed a hyperbolic trend. The black dotted line corresponds to the hyperbola fitting the data (equation in the inset).

In Figure 6, the Mean frequencies of the VGRF spectrum (i.e., those at the peak of the Gaussian curves) were plotted for each subject against the corresponding Peak amplitudes. Across the subjects, large peak spectral amplitudes corresponded to relatively lower spectral Mean frequencies. This was true when standing on Solid support as well, even if the amplitudes of the VGRF oscillations were small. The equations of the lines fitting the values of the Mean frequencies plotted against the Peak amplitudes are reported in Table 1 for each vision and support condition.

**Table 1:** Equations of the lines fitting the values of VGRF Mean frequency versus Peak amplitude in the four standing conditions.

**VGRF mean frequency vs. peak amplitude**	**Equation**	**R^2^**	***p***
EC foam	y = −1.95 x + 3.86	0.06	0.27
EO foam	y = −5.87 x + 4.12	0.10	0.13
EC solid	y = −98.8 x + 4.92	0.07	0.21
EO solid	y = −171.9 x + 5.04	0.10	0.13

Considering all vision and support conditions together, the scatter of the data suggested the possibility that a hyperbolic function could fit the relationship between the two variables, in the assumption that a common behavior underpins the VGRF oscillations across all subjects and all conditions. The hyperbolic fit through all data points shows that large peak amplitudes of the VGRF spectra were associated with low frequencies and vice versa. The equation of the function is reported in Figure 6.

### Relationship between VGRF spectrum and geometric variables (sway area and path length)

**Figure 7:**
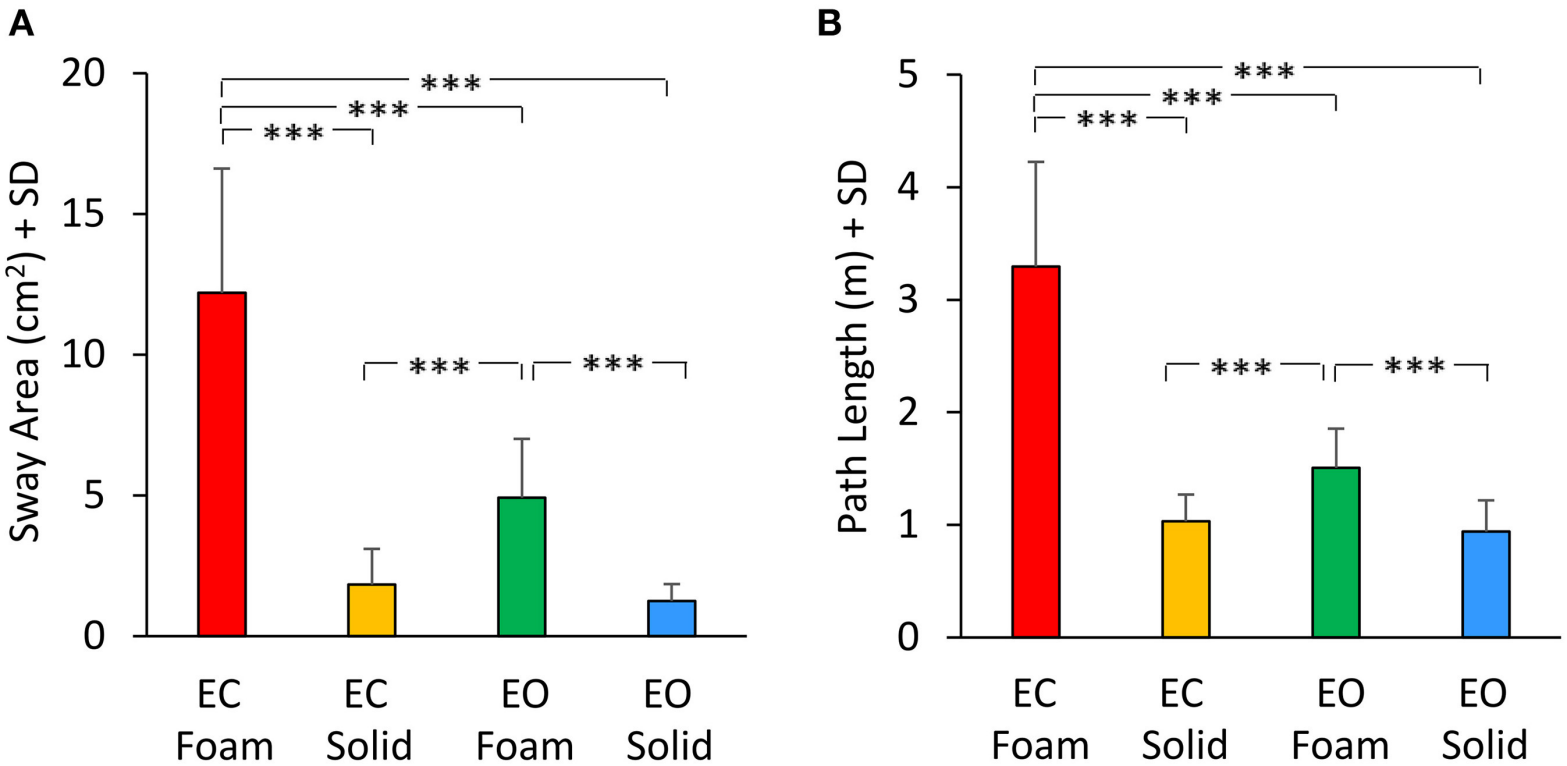
Geometric measures. Mean values of Sway Area **(A)** and Path Length **(B)** for each visual and base of support conditions. Both Sway Area and Path Length were greater with Foam than Solid support. The effect of vision was significant only with Foam. Asterisks indicate significant differences (***p < 0.001).

The mean values of Sway Area and Path Length are reported in Figure 7 for each vision and support conditions. Sway Area (Figure 7A) was greater with Foam than Solid support [main effect, *F*_(1, 22)_ = 190.67, *p* < 0.001, $\eta_{\text{p}}^{2}$ = 0.89] and with EC than EO [main effect, *F*_(1, 22)_ = 76.71, *p* < 0.001, $\eta_{\text{p}}^{2}$ = 0.78]. A significant interaction between vision and support conditions was found [*F*_(1, 22)_ = 64.41, *p* < 0.001, $\eta_{\text{p}}^{2}$ = 0.74]. There was a difference in Sway Area between EO and EC with Foam (*post-hoc, p* < 0.001) but not with Solid support (*post-hoc, p* = 0.38). Also for Path Length (Figure 7B) there was a significant difference between support conditions [Foam > Solid: main effect, *F*_(1, 22)_ = 204.54, *p* < 0.001, $\eta_{\text{p}}^{2}$ = 0.90] and between vision conditions [EC > EO: main effect, *F*_(1, 22)_ = 106.96, *p* < 0.001, $\eta_{\text{p}}^{2}$ = 0.83]. The interaction between vision and support conditions was significant [*F*_(1, 22)_ = 103.25, *p* < 0.001, $\eta_{\text{p}}^{2}$ = 0.82]. There was a difference between EC and EO in Path Length with Foam (*post-hoc, p* < 0.001) but not with Solid support (*post-hoc, p* = 0.48).

**Figure 8:**
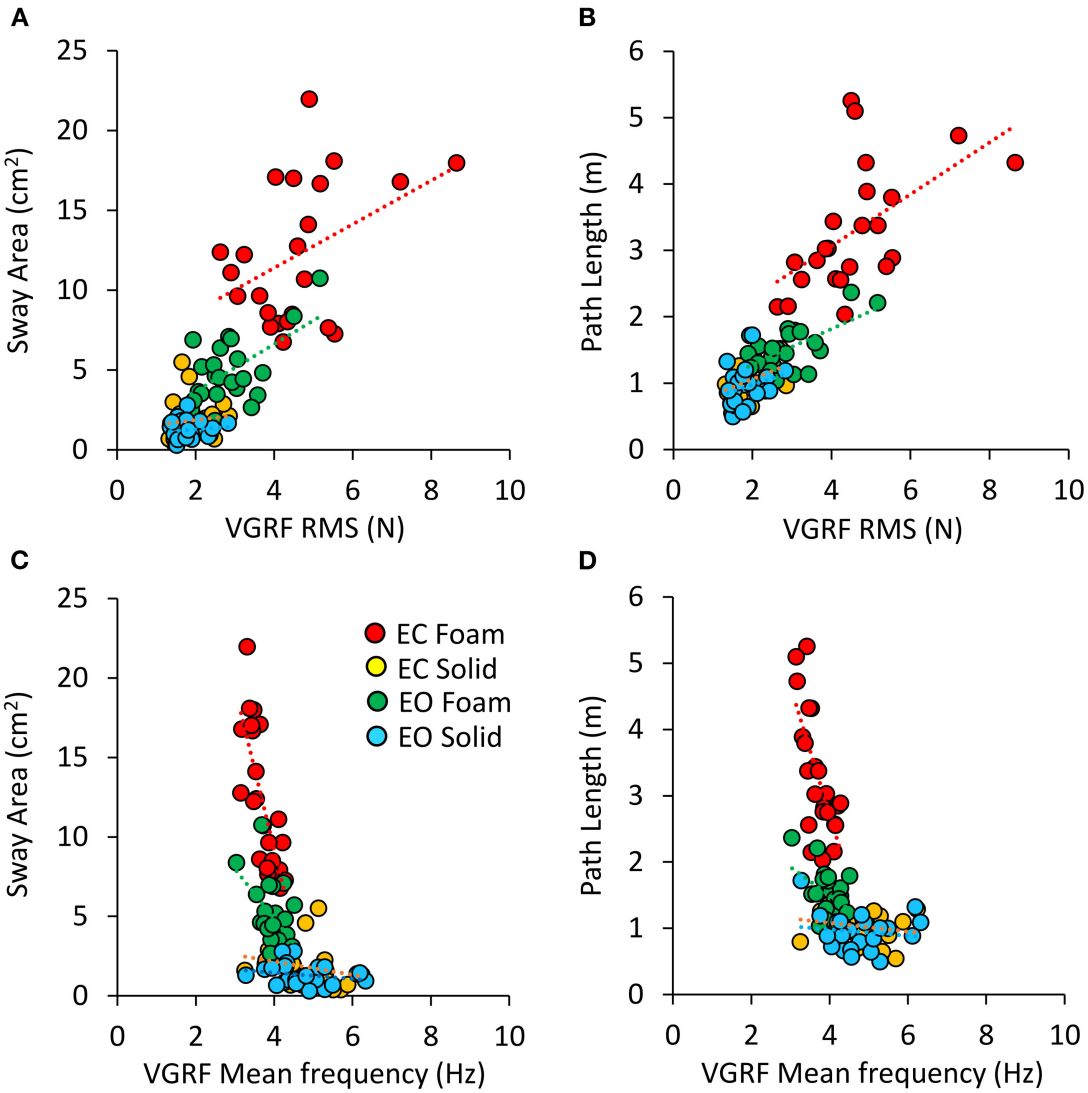
Relationship between geometric measures and VGRF RMS and Mean frequency. Sway Area of each subject was plotted against the corresponding value of VGRF RMS **(A)** and of VGRF Mean frequency **(C)**. On the right, Path Length was plotted against the corresponding value of VGRF RMS **(B)** and VGRF Mean frequency **(D)** (EC Foam, red; EC Solid, yellow; EO Foam, green; EO Solid, blue). The equations of the regression lines for each variable and condition are in Tables 2, 3.

There was a noticeable similarity between Figure 2A (showing VGRF RMS in the tested conditions) and Figure 7B. Therefore, in Figure 8, the values of Sway Area (A) and Path Length (B) have been plotted against the corresponding values of the VGRF RMS. In both cases, there was an obvious proportionality between the two variables, Sway Area and Path Length generally increasing across vision and support conditions with the increase in VGRF RMS. Sway Area and Path Length have also been plotted against the corresponding values of the prevalent VGRF frequency at the peak of the Gaussian curve (Figures 8C,D). Across the subjects, the larger Sway Area or Path Length, the lower the prevailing frequencies of the VGRF oscillations. All visual and support conditions contributed to these relationships. However, within each condition, the slope of the linear relationships was not equally strong, either with or without vision. The equations of the lines fitting the values of Sway Area or Path Length against both the VGRF RMS and the VGRF Mean frequency are reported in Tables 2, 3, respectively. Of note, even in the EC Foam conditions, the excursions of the CoP were limited, attesting a good postural control. The mean value across subjects of the maximal excursions was 2.6 ± 0.8 cm in the ML direction and 3.0 ± 0.7 cm in the AP direction.

**Table 2:** Equations of the lines fitting the values of Sway Area and Path Length versus VGRF RMS in the four standing conditions.

**Sway area vs. VGRF RMS**	**Equation**	**R^2^**	***p***
EC foam	y = 1.36x + 5.91	0.17	<0.05
EO foam	y = 1.48x + 0.67	0.34	<0.01
EC solid	y = 0.29x + 1.27	0.01	0.62
EO solid	y = 0.18x + 0.91	0.01	0.27
**Path length vs. VGRF RMS**			
EC foam	y = −0.39x + 1.5	0.32	<0.01
EO foam	y = 0.27x + 0.73	0.41	<0.01
EC solid	y = 0.24x + 0.57	0.22	<0.05
EO solid	y = 0.19x + 0.59	0.07	0.19

**Table 3:** Equations of the lines fitting the values of Sway Area and Path Length versus VGRF Mean frequency in the four standing conditions.

**Sway area vs. VGRF mean frequency**	**Equation**	**R^2^**	***p***
EC foam	y = −10.1x + 49.6	0.59	<0.001
EO foam	y = −3.11x + 17.3	0.25	<0.05
EC solid	y = −0.42x + 3.81	0.06	0.25
EO solid	y = −0.19x + 2.22	0.05	0.26
**Path Length vs. VGRF mean frequency**			
EC foam	y = −1.94x + 10.5	0.49	<0.001
EO foam	y = −0.42x + 3.19	0.16	0.06
EC solid	y = −0.07x + 1.34	0.04	0.34
EO solid	y = −0.05x + 1.18	0.02	0.53

## Discussion

Several past and recent studies have secured crucial information on the human gait thanks to the analysis of the vertical ground reaction force (VGRF) recorded during the stance phase of walking (52–55), where the GRF shows a range of orientations of major components, including horizontal (frictional) forces with the ground. Characteristics of the human upright stance have been addressed based on the analysis of the VGRF as well. However, the vertical force active on force sensors of the platform upon which subjects stand has been exploited almost exclusively in order to describe the excursions of the Centre of Pressure (CoP) on the horizontal plane (7, 56, 57). VGRFs have been recorded and analyzed sparingly, even if they are at the interface between the “postural control strategy” and the body sway measures deduced by the CoP excursions (58, 59). Looking at the VGRF with new eyes, however, has given direct, intriguing perspective on the control of body balance by standing subjects and on the plan of action to maintain the upright posture under different sensory and support surface conditions (60).

Here, we have found that: (a) the amplitude (RMS) of the VGRF oscillations is larger on the compliant that hard support surface; (b) there is a modest correspondence between VGRF RMS and amplitude of Sway Area and Path Length of the CoP; (c) the oscillations of the VGRF have a dominant frequency around 4–5 Hz in both support conditions, but the frequency range is larger on hard than compliant support; (d) vision compared to no-vision reduces the amplitude of the VGRF oscillation on the compliant but not hard surface; (e) vision does not affect the frequency of oscillation (on either supports); (f) in general, the higher the oscillation frequency, the lower the amplitude of the VGRF oscillations. We have also confirmed that the VGRF prevalent frequencies are well beyond the oscillation frequencies of the CoP excursion in either the anteroposterior or mediolateral direction.

### Amplitudes and frequencies of the VGRF: The effect of the support base

The oscillations of the VGRF around the body weight are of moderate amplitude having a RMS value of about 5 N (or 0.5 kg) on compliant support, and even smaller on the hard platform, about 2 N (or about 0.2 kg). Then, since subjects were standing on both feet and actively controlling the postural muscles, particularly on the compliant support, a reasonable, supposition is that the vertical force repeatedly increased underneath one and decreased underneath the other foot in the mediolateral direction, dictated by the “hip strategy” implying a non-negligible CoP SD in the ML direction as shown by Sozzi et al. (28). Conversely, in the anteroposterior direction, when sway is under the control of the muscles around the ankle, the vertical force would decrease below the toes and increase at the heels or vice versa during the ankle dorsiflexions and plantarflexions. This is in keeping with the larger CoP SD in the frontal than in the sagittal plane (28). In this light, the frequency of the oscillations of the VGRF would depend on the rate of change in the decreasing and increasing phases of the rhythmic motion of the body and its segments in the vertical direction. These would produce the resulting vertical force changes due to the torques exerted by the activated muscles on the support surface (38).

The largely superimposable VGRF oscillation frequency values at the peak of the spectrum profile (as identified by the Gaussian curve fitted to it) when standing on both the hard and the compliant support bases was an unexpected finding. This occurred in spite of much larger force oscillations (more than ten times) on the compliant than hard support, quantified by the root mean square of the time series (RMS, or “signal strength,” in N). There was a significant difference in the frequencies of the VGRF between Foam and Solid support indicating a consistent behavior among the subjects in each of these two conditions. But the difference was moderate, because the mean frequency of VGRF oscillations at the peak of the Gaussian curve was only about 13% larger on hard than compliant surface. Instead, a consistent difference in the power spectrum profile was shown by the frequency range at both sides of the peak of the spectrum, whereby the Gaussian curve had a low peak and broad slope (a larger SD) with hard support, indicating that VGRF frequencies were represented in a larger range of values. These data are in keeping with a previous report by Oggero et al. (33). The highest VGRF amplitudes were found in a range between 2 and 8 Hz, with pronounced peak around 4 Hz, also by Günther and Wagner (43) in subjects standing quietly on a solid base of support (see their Figure 7).

The most represented VGRF frequencies would speak in favor of a basic mode of muscle activation underpinning standing (that we have named here the “postural rhythm”), common to hard or compliant bases of support (61). The small differences in the prevailing oscillation frequencies between compliant and hard support can be reasonably attributed to the mechanical properties of the foam pad (featuring a visco-elastic structure), which introduces a time delay between the development of the muscle torques and the recorded force, allowing a more extended joint excursion than on hard support and leading to lower frequency of the continuous rhythmic muscle action (37). On the hard support, the VGRF oscillations would be much smaller in amplitude because weak muscle activity (in time and space) would be amply sufficient for body balancing. Hence, the control of upright posture would result primarily in minimal ankle motion with stiff knee at a somewhat higher prevailing VGRF frequency (62), while the larger instability on foam would produce more hip and knee than ankle motion and slightly lower peak frequencies of force oscillation (63). We should add that standing on either support did never produce instability or kind of stumbling, forcing the subjects to lift a foot or move an arm.

### The effect of vision

The comparable VGRF frequencies when standing with and without vision were unanticipated. It is known that the amplitude of the sway of the CoP excursions is definitely larger with EC than EO (64) and larger on compliant than solid support [see e.g., (4)]. The median frequency of the CoP excursions on the horizontal plane (both AP and ML) is higher with EC than EO as well (4, 65, 66). However, the mean frequencies of the VGRF oscillations showed no major effect of vision. If anything, frequencies were seemingly slightly higher with EO than EC. The lower frequency at the peak of the Gaussian curve on compliant than hard support was also independent of vision. It seems that access to vision does not modulate the frequency of the muscle actions producing the VGRF oscillations. While this comes as no surprise when standing on hard support, where the CoP excursions and sway area are often superimposed with or without vision (65, 67) and where the effect of vision is larger for narrow feet positions (68), the larger body sway on Foam EC than EO is independent of the VGRF frequency. Rather, vision largely modulates the amplitude of the VGRF oscillations (the RMS) concurrently with the effects on body sway (Figures 1, 8A,B). This is also suggested by the shape of the relationship between VGRF frequency and VGRF amplitude, featuring a hyperbolic association, where the amplitude of the oscillations ranged from near zero N^2^ (on hard support, regardless of vision) to 0.16 N^2^ (without vision), while the frequency of the oscillations ranged from about 4 to about 6 Hz (Figure 6).

Hence, vision seems to hardly affect the basic rhythm of the muscle actions but to limit their vigor. Under different conditions (standing on a translating platform), the activity of many postural muscles rapidly diminished when vision was allowed, leading to reduction of body segments' swinging back and forth, without effect on the latency of the responses (69, 70). Similarly, standing on a very mobile support was not possible without vision (71). No direct question has been posed here or methods employed in order to assess the consistency of the frequency of the descending command when standing on hard or compliant support, with or without vision. However, we would remind here the conclusions by Jacobs et al. (72), who found no significant differences in cortico-muscular coherence, as a sign of cortical involvement in human standing balance, when comparing standing with eyes open to standing with eyes closed and standing on hard vs. compliant surface. We would speculate that the increased amplitude of the VGRF oscillations with EC on compliant support would be a sign that the vestibular reflexes take the lead under this condition (73–75), without affecting the basic, rhythmic pattern set by the cortical control of stance. As a side note, we would remind that the dichotomy vision/no-vision in postural control has to be still convincingly demonstrated. Schmid et al. (76) cast doubt on the simple, conservative hypothesis of two fundamentally different balancing strategies. Rather, the body seemed to exhibit a continuous mode of balancing patterns as a function of visual acuity.

### The postural rhythm

It is remarkable that the muscle actuators of balance control and corrections are substantially rhythmic within a restricted cadence range, and the rhythm is little affected by support compliance. Here, the frequency range of the VGRF is compatible with values found by others (33, 43, 77–79). Around these prevailing frequencies, a wider range of lower and higher oscillations is shown by the tails of the Gaussian curve, broader on hard than compliant support.

Interestingly, the prevailing frequency values are close to the human body resonant frequency, assumed to be in the range of 3 to 7 Hz (78, 80–83). The continuous variations in the VGRF oscillation frequency would be basically tuned to the body resonant frequency and to the capacity of the muscles to produce the corrective balance reactions. These would be in turn associated in some respect to their twitch time course. The torque exerted by the muscles around the ankles, in standing subjects voluntarily contracting their leg and foot muscles in a reaction time mode, has a duration of around 250 ms (84) similar for both toe-down and toe-up actions. Others have found that a figure of 3–4 bursts per second is compatible with an “intermittent” control (85, 86). In principle, then, the lowermost frequency would be set by the time duration of the mechanical action of the muscle twitch time course (15, 16).

For example, when standing on the compliant support, the toe-up action leading to a forward body displacement would be promptly counterbalanced by the toe-down action, thereby producing a peak in the VGRF at a frequency around 4 Hz. If the two legs would operate (at least partly) independently, the slope of the Gaussian curve with a peak around 4 Hz would easily materialize. The same basic pattern of control would be exploited for standing on hard support. As said, however, on hard support the borderline frequencies are relatively more represented, subjects showing smaller spectral amplitude at (relatively) lower and higher oscillation frequencies. When the support is hard, the postural rhythm is tuned to frequencies continuously variable in a wider range than on compliant support, including frequencies from about 3 Hz to about 8 Hz. It is expected that low frequencies occur on hard support (15), because longer “stable” periods would occur on a safe and easy stance. On the other hand, notably so, frequencies around 7–8 Hz have been recorded on the bare platform on vestibular galvanic stimulation (87). The higher small-amplitude frequencies on hard support would speak for small-amplitude sub-tetanic contractions, with production of force ripples at shorter intervals. These frequencies are still below the production of fused muscle contractions but would tend to favor some tonically maintained levels of force in the slow-twitch postural muscles [having long twitch time course, see e.g., (88)]. In all cases, the higher VGRF oscillation frequencies appear to find their upper limit in the body resonant frequency, hence they are seemingly appropriate to produce the necessary sway corrections with the minimal possible effort. The significantly lower prevailing frequency and the more limited frequency range with compliant than hard support (33) would be explained by the minor fluctuating changes in the resonant frequency observed under relaxed than stiff posture, likely due to variable bending of lower limb joints (78, 89).

Interestingly, continuous antagonist muscles co-activation (likely featuring small, high frequency VGRF oscillations) is linked to poor postural steadiness in the elderly, and is accompanied by enhanced stiffness and larger body sway (90–92). Co-contraction has been associated with increased CoP excursion frequency and shown to be not necessarily helpful for stabilization of upright posture (93, 94).

### The oscillation frequency of the VGRF are not represented in the frequencies of CoP oscillations

One wonders why the prevailing frequency of VGRF variation is so different from those of the excursion of the CoP, where the 98% of the power spectrum is normally comprised below 2 Hz (4, 5, 95), and where the median frequency attests itself at about 0.3 Hz with EC and 0.15 Hz with EO (4, 77, 96, 97). This comes as no surprise, though, because the CoP location is the resultant of the torques acting onto various positions of the CoP on the platform and can theoretically have infinite solutions. Suffice it to say that, if the upper segments of the body (e.g., trunk and head) move with their own speed (probably low) back and forth or sidewise or in any directions of the space, their displacement would affect the horizontal position of the Centre of Mass (CoM) and the CoP excursion but not necessarily the VGRF frequency. These motions would have an effect on the CoP oscillation frequency according to their own displacement speed, which is in turn dependent on the force of the muscles acting on the mass of those segments (60, 98).

While the twitch time course of a postural muscle can be of the order of several tenths of ms, the elicited movement of the body can be fast (but definitely not as fast as the twitch time-course) or extremely slow as when the postural conditions impose a quasi-isometric muscle contraction. If anything, our findings support the view that modeling body sway with an inverted pendulum hardly provides for a useful generalization of the balance strategies (5, 22, 99). Fluctuations in ankle, knee and hip joints participate in upright stance adjustments (100). These fluctuations may not be obvious in the frequency spectrum of the CoP displacement, but frequencies around 4–8 Hz have been recently attributed to angular fluctuations in the leg joints (20, 21). The rhythmic contributions of the knee joint to the mechanical dynamics of the standing body would mostly appear as vertical motions of the CoM with corresponding frequency content, in keeping with our findings obtained by spectral analysis of the VGRF. It has been recently shown that continuous motions of ankle, knee and hip joints induce coordinated shifts of the line of action of the ground reaction force above or below the CoM, producing changes in both the VGRF and CoP oscillations (92, 98, 101). Under different conditions (locomotion), very small displacements of the CoP can produce very large, slow displacements of the CoM by minimally changing the CoP instantaneous position and the direction of the VGRF vector, as occurs during walking and gentle turning (102).

Interestingly, a minor increase in the CoP frequency spectrum in both the frontal and the sagittal plane between 3.5 and 6 Hz frequency had been described by Krafczyk et al. (103) in people with psychogenic vertigo standing on a compliant surface. This had been attributed to co-activation of anti-gravity muscles, and interpreted as a shift from automatic to conscious control. An effect of surface compliance was noted at high frequencies (4 to 7 Hz) in the CoP spectrum in medio-lateral direction by Singh et al. (32) and attributed to high frequency activity of the muscles controlling the lateral body sway. This possibility merits further investigation, and would require analysis of the VGRF oscillations together with the analysis of the CoP frequencies in a larger range beyond the usual upper limit of 2–3 Hz [see (4)] that is often chosen because most of the CoP spectrum is normally comprised below 2 Hz.

### Relationship of VGRF oscillation amplitudes with the common geometric markers of stance

It is remarkable that the spectral amplitudes measured by the RMS or the spectral amplitudes at the prevailing frequency (as seen in Figures 2, 6, respectively) almost mirror the pattern of mean Sway Area and Path Length in the corresponding vision and support conditions (Figures 7A,B). It would not be wise to assume a strict cause-effect association between these variables, because there is an ample variation across subjects (Figure 8). A significant association is present only on the compliant support (Figures 8A,B), where both VGRF spectral amplitudes and amplitude of body sway, independently, would express the difficulty of standing on the compliant surface. Conversely, on the hard support, the association disappears. Here, the coefficients of determination are weak and not significant, indicating that important confounding factors interfere. Therefore, the amplitude of the spectrum of the VGRF at its maximally represented frequency of oscillation cannot be considered a proxy for body oscillation, particularly when standing on hard support (59).

Of note, the stability boundary (where the subjects lean as far as possible in all directions of the horizontal plane without losing stability) has been shown to be just smaller on foam than on hard surface, and the velocity of the CoP excursion to be higher (104). We would also add that the largest excursions of the CoP in the horizontal plane in our subjects were largely within the stability boundaries, regardless of vision condition and support feature. These have been found to be about 10 and 7 cm in ML and AP, respectively, slightly greater with than without vision and on hard than solid support (104). In our hands, CoP never really came close to the boundaries, because its maximal excursions hardly ever exceeded 2–3 cm in the most critical stance condition (Foam, EC) (as in Figure 1).

### Proprioception subserves postural adjustments on foam

The use of a compliant standing surface has been often motivated by the attempt to disturb, attenuate or nullify the proprioceptive information (105–110). Such disturbing effect would be stronger in older persons (35) even if foam was not discriminant in all studies (111). Teasdale et al. (112) showed that removal of one sensory input in isolation (e.g., standing on foam) was not sufficient to differentiate between elderly and young adults, and explained this as the result of compensation by the remaining sensory sources. We do not believe that a compliant surface excludes or disrupts proprioception. There is no reason why proprioception should be attenuated or muddled when standing on foam. Muscle spindles are very sensitive to rapid muscle stretches [see (113)], particularly so for the primary terminations of the large Ia fibers (114). Conversely, the secondary terminations of the group II afferent fibers are sensitive to elongation (less so to its velocity). Both receptors produce clear cut and powerful homonymous and heteronymous motor reflexes (115–117) and afferent volleys reaching distant spinal centres (118) and supra-spinal centres as well (119–122). The findings by Chiang and Wu (123) support a similar role of proprioceptive information when standing on hard or compliant surface. In both circumstances, when the platform upon which subjects stood was rotated, the angular velocity of the ankle joint was similar, and the latencies of the evoked reflex responses were just minimally increased by standing on foam. If anything, it might be crudely schematised that standing on the complaint surface exploits a powerful input along the Ia fibers, while standing on the hard surface favors group II fiber activity (124–127). Hence, both spindle afferent inputs from the muscles of the foot (116) and of those around the ankle would contribute their information to a common feedback control of stance on hard and compliant surface (17, 128, 129). Of course, these inputs would not be the same under both support surface conditions (130), and the reasonable conclusion is that standing on a compliant or on a hard surface are two different tasks with a common task goal. But this is kind of a truism. We would rather suggest that one of the reasons for the broader VGRF frequency spectrum profile on hard support might depend on the prevalent input of the group II spindle fibers, which are sensitive to position more than velocity of body displacement and able to elicit reflex responses on both the ipsi- and contralateral leg muscles (117).

### The interaction between sensory inputs and motor commands

The modest (but significant) difference in the frequency range of the oscillations in the VGRF on hard and compliant support would speak for a common sensory input to the brain and a common feedback operation. Whereas, the much larger peak amplitude of the spectrum for compliant than hard support surface would be explained by the strong and rhythmic activity of the muscles involved in the balance correcting reactions (131), in turn connected with the need for more vigorous contractions. On compliant surface, the feedback would recruit several body muscles (20, 75, 118, 132–134) and operate together with anticipatory activations (135, 136). This interaction would be modulated by the sensory inputs and efference copies connected with the continuous body sway and muscle actions, respectively (137).

The information from the cutaneous receptors of the foot sole may fine-tune the VGRF frequencies. Recently, these receptors, with focus on their role in posture, have received attention by various groups [(138–141); see (142)]. Their contribution is different between hard and compliant surface, not least because in the former case a continuous stimulation of these receptors occurs over the foot sole, while in latter case sway could help periodically release skin compression to reactivate mechanoreceptors (143). The larger contact with the compliant than hard support surface might enhance the feedback control from the feet [see (144)].

The control mode would possibly imply both a temporal check during the major reactions to destabilization and an amplitude check during the unavoidable counterbalancing phase (145). Balancing and counterbalancing would require alternate actions transmitted to the support surface by reciprocal activation of, say, the triceps of one body side and the pretibial muscles on the other side when the destabilization occurs in the roll plane and activates muscles in addition to those about the hip (146). In the case of the pitch plane, this alternation would occur in rapid succession and simultaneously on both sides (146). The frequency of the descending commands might be similar in both circumstances. Conversely, the “safer” condition of standing on a hard support with feet apart allows an easier selection of an optimal time- and space-dependent strategy aimed at minimizing muscle activation (147, 148), under conditions where minor but precise activation of the intrinsic foot muscles can be very effective (18, 149).

In general, we would admit that different subjects across the various standing conditions choose slightly different standing modes, by modulating both the amplitude of sway and the frequency of VGRF. The hyperbola fit to all subjects' VGRF mean frequency against the VGRF peak amplitude (Figure 6) would suggest a common pattern, whereby oscillations at predominantly high frequency values (on hard support) are associated to small-amplitude VGRF values, while lower frequencies (particularly on compliant support) are associated to large-amplitude force oscillations. A similar pattern is shown by the plot of Sway Area vs. VGRF frequency across subjects, where the higher VGRF frequencies are associated with small CoP displacement (be it Sway Area or Path Length) and the opposite is true for low frequencies. Of course, the data points corresponding to different vision and support conditions cluster around different regions of the hyperbola, but we would be surprised of an unlike distribution. In a sense, this hyperbolic pattern indicates that the intensity of the effort implicit in standing is broadly constant in a population of young healthy subjects. This intensity is either allocated to the rhythmicity of the production of the active muscle forces or to the control of the amplitude of the contraction, therefore of the body movements allowed. In this context, vision and support compliance stipulate their constraints in compliance with the properties of the neural control (41). The hyperbola also shows that VGRF oscillation frequencies have a limit below 3 Hz in all subjects on both compliant and hard support. Thus, it would be safe to state that subjects are never quasi-static, suggesting that a control programme is always running.

### Limitations

The number of participants was limited and the study was restricted to young healthy adults. In addition, we considered only one test per condition (vision and support) per subject. Even so, where vision and support conditions produced significant differences in VGRF (for VGRF RMS, Peak amplitude and Mean frequency, SD of the Gaussian fit) and in the geometric measures, the effect size was greater than the minimum effect size set by our sample of 23 participants. Absence of trial reiteration was justified by the great adaptation that we recently described in the frequency values of the CoP excursions in repeated successive trials (28). However, the long acquisition epoch (90 s) would permit to be confident on the values of the recorded patterns. As a further limitation, the participants to these experiments were not instructed to stand “as still as possible” (150) under all test conditions. We were concerned that that instruction would have favored a stiff attitude and a possible stumbling reaction when subjects felt unstable on foam. The instruction was the same on hard support. We cannot deny the possibility that this loose specification enhanced the inter-individual variability and the idiosyncratic characteristics of postural sway (151). We did not manipulate the between-feet distance, either (152), or the characteristics of the foam pad (38, 153), because many volunteers were not ready to repeat the trials in different days. Further, our platform did not allow separate recording of the VGRF of the right and left foot [see (26)] that would have helped identify the role of the inter-leg coordination in setting the postural rhythm. Moreover, there has been no optokinetic (or otherwise) direct recording of the body segments' motion. This would be necessary because most recent models of human stance are based on motion in the sagittal plane, but standing on a compliant surface is more complex. Moreover, ECG/EMG recording that might have helped disclose the contribution of the heart beat to the VGRF oscillations (21, 154, 155). This would be relevant in people with some forms of a central nervous system pathology (156). These experiments are needed, and will likely offer independent ways to confirm the suggestions put forward so far.

### Conclusions and perspectives

Together with imposed perturbations, support surface manipulations provide an additional tool to investigate how sensory and motor signals are integrated to control balance during standing. The present findings have addressed the features of the VGRF under hard and compliant surface in young healthy subjects, and are preliminary to more extended research. The foam support produces mechanical perturbations and sensory inflows that elicit active compensatory responses achieving body stabilization with the least effort. These mechanical responses are quite rhythmic on foam, and still rhythmic on hard support, even if less ample and regular. Under both circumstances, though, a prevalent frequency in the VGRF emerges, close to the natural body resonant frequency. Vision does not affect the peak VGRF frequency. Anyhow, vision helps strongly reduce the amplitude of the oscillation frequencies and probably restrains sway by focussing on the anticipatory control of upright body orientation [(157, 158); see (159)], without necessarily “recruiting” new, special frequencies of postural muscle activation as appreciable in the VGRF oscillation pattern.

At variance with the traditional measures of CoP excursions in the horizontal plane, the VGRF is directly affected by the postural muscle action. The information provided by the analysis of the VGRF is roughly superimposed to that of the geometric measures (length and surface covered by the CoP trajectory), if the *amplitude* (the RMS) of the VGRF oscillations is compared to the traditional geometric measures. But, VGRF oscillations *frequencies* are instead only loosely related to the geometric measures. It seems that the VGRF oscillations represent a basic rhythm that creates the conditions for the successful action of superimposed focused muscle activations, responsible for the emergence of stabilizing torques (98). Since the CoM would not always oscillate in precisely the vertical direction, the horizontal component of the GRF would create the horizontal translations of the CoM (101).

We suggest that future analysis of the frequency pattern of VGRF oscillation and of the EMG bursts of the postural muscles would help address the origin and the modulation of supra spinal control, the reflex and intrinsic joint stiffness components participating in the control of posture [see (160)], and the physiological mechanisms responsible for balance modifications (161). The effect of clinically relevant sensory and motor deficits [e.g., otolith dysfunction, (162, 163)] might be usefully addressed by adding analysis of the prevailing VGRF oscillation frequencies to those of the geometric measures. Further, since adaptation over successive standing trials has been recently described (5), it will be relevant to investigate whether and how VGRF can be independently modulated by the adaptation process to enhance postural stability. We hope that the present structural approach can have diverse applications in the field of balance control. It might help classify and understand postural states in various populations with balance problems, as has been done already with older persons and Parkinson's disease patients (29, 31, 77, 164). We would speculate that reduction in the nerve conduction velocity in neuropathies or muscle weakness of various origin might find convenient markers in the VGRF frequency or RMS, respectively [see (165), for a discourse on this possibility]. Further research is needed to connect the present findings to possible sources of potential disorders in the postural control system.

## Data availability statement

The raw data supporting the conclusions of this article will be made available by the authors, upon reasonable request.

## Ethics statement

The studies involving human participants were reviewed and approved by Istituti Clinici Scientifici Maugeri IRCCS, Approval Number #2564-CE. The patients/participants provided their written informed consent to participate in this study.

## Author contributions

MS conceived the idea for the study. SS performed the recruitment of participants and the collection of data. SS and MS performed the data analysis and drafted the article. M-CD revised it critically for important intellectual content. All authors approved the submitted version.

## Funding

The funding for this study was provided by the ICS Maugeri SB, through the Ricerca Corrente program of the Italian Ministry of Health.

## Conflict of interest

The authors declare that the research was conducted in the absence of any commercial or financial relationships that could be construed as a potential conflict of interest.

## Publisher's note

All claims expressed in this article are solely those of the authors and do not necessarily represent those of their affiliated organizations, or those of the publisher, the editors and the reviewers. Any product that may be evaluated in this article, or claim that may be made by its manufacturer, is not guaranteed or endorsed by the publisher.
